# Clinical Profile and Risk Factors for Severe COVID-19 in Hospitalized Patients from Rio de Janeiro, Brazil: Comparison between the First and Second Pandemic Waves

**DOI:** 10.3390/jcm12072568

**Published:** 2023-03-29

**Authors:** Luciane Almeida Amado, Wagner Luis da Costa Nunes Pimentel Coelho, Arthur Daniel Rocha Alves, Vanessa Cristine de Souza Carneiro, Otacilio da Cruz Moreira, Vanessa Salete de Paula, Andreza Salvio Lemos, Larissa Araujo Duarte, Elisa Gouvea Gutman, Fabricia Lima Fontes-Dantas, João Paulo da Costa Gonçalves, Carlos Henrique Ferreira Ramos, Carlos Henrique Ferreira Ramos Filho, Marta Guimarães Cavalcanti, Marisa Pimentel Amaro, Rafael Lopes Kader, Roberto de Andrade Medronho, Dmitry José de Santana Sarmento, Soniza Vieira Alves-Leon

**Affiliations:** 1 Laboratory of Technological Development in Virology, Oswaldo Cruz Institute/Fiocruz, Rio de Janeiro 21040-900, Brazil; 2 Laboratory of Molecular Virology, Oswaldo Cruz Institute/Fiocruz, Rio de Janeiro 21040-900, Brazil; 3 Real Time PCR Platform RPT09A, Laboratory of Molecular Biology and Endemic Diseases, Oswaldo Cruz Institute/Fiocruz, Rio de Janeiro 21040-900, Brazil; 4 Laboratory of Translacional Neurosciences, Biomedical Institute, Federal University of the State of Rio de Janeiro-UNIRIO, Rio de Janeiro 22290-240, Brazil; 5 School of Medicine, Federal University of Rio de Janeiro, Rio de Janeiro 21941-901, Brazil; 6 Department of Pharmacology and Psychobiology, Roberto Alcântara Gomes Institute Biology (IBRAG), Rio de Janeiro State University (UERJ), Rio de Janeiro 20551-030, Brazil; 7 Unit of Intensive Treatment, Clementino Fraga Filho University Hospital, Federal University of Rio de Janeiro, Rio de Janeiro 21941-901, Brazil; 8 Epidemiology and Evaluation Service, Clementino Fraga Filho University Hospital, Federal University of Rio de Janeiro, Rio de Janeiro 21941-901, Brazil; 9 Post-Graduate Program in Infectious and Parasitic Diseases, School of Medicine, Federal University of Rio de Janeiro, Rio de Janeiro 21941-901, Brazil; 10 Department of Oral Diagnosis, School of Dentistry, State University of Paraíba, Araruna 58429-500, Brazil; 11 Department of Neurology, Reference and Research Center for Multiple Sclerosis and Other Central Nervous System Idiopathic Demyelinating Inflammatory Diseases, Clementino Fraga Filho University Hospital, Federal University of Rio de Janeiro, Rio de Janeiro 21941-901, Brazil

**Keywords:** COVID-19, risk factors, epidemiology, epidemic waves

## Abstract

Since COVID-19 was declared a pandemic, Brazil has become one of the countries most affected by this disease. A year into the pandemic, a second wave of COVID-19 emerged, with a rapid spread of a new SARS-CoV-2 lineage of concern. Several vaccines have been granted emergency-use authorization, leading to a decrease in mortality and severe cases in many countries. However, the emergence of SARS-CoV-2 variants raises the alert for potential new waves of transmission and an increase in pathogenicity. We compared the demographic and clinical data of critically ill patients infected with COVID-19 hospitalized in Rio de Janeiro during the first and second waves between July 2020 and October 2021. In total, 106 participants were included in this study; among them, 88% had at least one comorbidity, and 37% developed severe disease. Disease severity was associated with older age, pre-existing neurological comorbidities, higher viral load, and dyspnea. Laboratory biomarkers related to white blood cells, coagulation, cellular injury, inflammation, renal, and liver injuries were significantly associated with severe COVID-19. During the second wave of the pandemic, the necessity of invasive respiratory support was higher, and more individuals with COVID-19 developed acute hepatitis, suggesting that the progression of the second wave resulted in an increase in severe cases. These results can contribute to understanding the behavior of the COVID-19 pandemic in Brazil and may be helpful in predicting disease severity, which is a pivotal for guiding clinical care, improving patient outcomes, and defining public policies.

## 1. Introduction

Severe acute respiratory syndrome coronavirus 2 (SARS-CoV-2) has rapidly infected millions of people around the world since its first detection in Wuhan, China, in December 2019 [1,2], quickly becoming an emerging health threat [3], and on 11 March 2020, the World Health Organization (WHO) declared it as a pandemic. In Brazil, the first case of coronavirus disease 2019 (COVID-19) was reported on 25 February 2020 [4]. Since then, Brazil has been one of the countries most affected by the COVID-19 pandemic, facing more than 34 million confirmed cases and 685,000 confirmed deaths by 30 September 2022 (according to the JHU CSSE COVID-19 data). 

Brazil is an upper-middle-income country with 210 million inhabitants in a large territorial area, with a substantial socioeconomic heterogeneity between its five macroregions (north, northeast, central–west, southeast, and south). The SARS-CoV-2 infection and disease burden have been highly variable across the country, probably due to existing regional disparities in access to and quality of health services, affecting the most vulnerable socioeconomic groups in the population to a greater extent [5,6]. COVID-19 cases were initially concentrated in the large metropolitan areas, such as São Paulo and Rio de Janeiro, spreading from state capitals to towns [7]. At the beginning of 2021, after approximately a year of the pandemic, a second wave of COVID-19 emerged with a rapid spread of a new SARS-CoV-2 lineage of concern, named lineage P.1, which had different features to the first wave in Brazil [8]. 

Recent studies have reported that Brazil faced a second wave that was more severe than the first one [9,10]. Nevertheless, previous studies have reported the general epidemiological profile, clinical characteristics, and outcomes of hospital-admitted patients with COVID-19, based on epidemiological data available in public dataset systems, in Brazil [9,10]. There is a paucity of analysis of the heterogeneity of the pandemic focusing on cohorts of critically ill patients infected with COVID-19 in Rio de Janeiro, a large metropolitan area in the southeast of Brazil. 

COVID-19 is typically characterized by viral pneumonia symptoms such as fever, fatigue, dry cough, anosmia, and headache, which can progress to acute respiratory distress syndrome (ARDS) [11,12]. Due to the ubiquitous distribution of the main viral entry receptor, the angiotensin-2-converting enzyme (ACE2), SARS-CoV-2 can cause a systemic disease, with possible involvement of the heart, liver, pancreas, kidneys, and central and peripheral nervous system, as well as the immune system [13,14]. 

Currently, several drugs are considered promising therapeutic agents for COVID-19 and have already been included in the treatment guidelines [15,16]. Among the different therapeutic agents evaluated for managing COVID-19, the most common that are registered for COVID-19 clinical trials are antivirals, monoclonal antibodies, and drugs modulating the renin–angiotensin system [17]. The FDA has authorized antiviral medications to treat mild to moderate COVID-19 in people who are more likely to become very unwell, including nirmatrelvir with ritonavir, remdesivir, and molnupiravir [15]. Remdesivir, an RNA-dependent RNA polymerase inhibitor (RdRp), which was the first approved drug for the treatment of SARS-CoV-2 infection, must be administered intravenously [18]. This implies that remdesivir administration requires a medical setup, which is not convenient for nonhospitalized patients. This problem was soon addressed by the development of molnupiravir, an orally bioavailable antiviral drug capable of inhibiting the replication of SARS-CoV-2 by inducing lethal mutagenesis and escaping viral proofreading activities. In vitro and in vivo studies have demonstrated potent antiviral activity of molnupiravir against SARS-CoV-2, and clinical trials have confirmed the good bioavailability, safety, and tolerability of this drug [19].

Since the onset of the COVID-19 pandemic, several vaccines have been granted emergency-use authorization. These vaccines have reduced COVID-19-related mortality rates, severe cases, and lengths of hospital stays in many countries [20,21,22]. However, the emergence of SARS-CoV-2 variants, mainly those that are considered variants of concern (VOCs), raise the alert for potential new waves of transmission, vaccine breakthrough infections, as well as the potential increase in pathogenicity [23]. The VOCs are associated with increased transmissibility or virulence, reduced neutralization by antibodies obtained through natural infection or vaccination, and an ability to avoid detection [24]. Therefore, it is essential to continue researching the epidemiology and molecular biology of this virus to improve therapeutic and vaccine efficacy, as well as to prevent an increase in transmission. 

Additionally, the actual vaccine effectiveness depends also on the vaccinated population and immunological status [25]. It was noted that there was high heterogeneity in vaccine effectiveness (VE) against SARS-CoV-2 infection among fully vaccinated individuals [26]. Less vaccine effectiveness was observed in the elderly population (VE = 83.8%) [26], who are more susceptible to infections and have poorer responses to vaccination due to immunosenescence and comorbidities [27,28,29,30]. Thus, it is worth noting that vaccination cannot eliminate the risk of infection [27]. Therefore, beyond vaccination, more measures need to be implemented to reduce the severe outcomes related to these infections.

Considering the actual scenario of COVID-19, knowledge of the baseline characteristics and outcomes of critically ill patients is crucial for better management of the disease and development of effective treatment strategies. Therefore, in this paper, we described and compared the demographic characteristics and clinical data of critically ill patients infected with COVID-19 hospitalized in Rio de Janeiro during the two waves of COVID-19. The main characteristics evaluated were symptoms, comorbidities/risk factors, data on clinical management, respiratory failure, and patient mortality. In this context, this study may be helpful to understand whether the high mortality risk of COVID-19 was also caused by an inadequate response of the health care system in the early stages of the pandemic. This information can also be used as a starting point in decision making in public health policies in other future COVID-19 “waves” worldwide. 

## 2. Materials and Methods

### 2.1. Study Population and Data Collection

This is a retrospective, descriptive study of hospitalized patients, with laboratory-confirmed COVID-19, who were admitted to Clementino Fraga Filho University hospital (HCFF) from Rio de Janeiro, Brazil, which is one of the referral centers for COVID-19. This work was approved by the Brazilian Ethics Committee (CONEP, CAAE 33659620.1.1001.5258). Participants were recruited using convenience sampling. The inclusion criteria were all consecutive severe and non-severe patients with laboratory-confirmed COVID-19 who signed the consent forms (or their guardian/legal representative did) and who were admitted to this hospital from July 2020 to October 2021. 

Pregnant women were not included in this study because there was no availability of such patients at the Hospital Universitário Clementino Fraga Filho, as they were referred to another reference hospital of the university (Maternity School).

Severe patients were defined as patients admitted to the intensive care unit (ICU) who required mechanical ventilation and had a sequential organ failure assessment (SOFA) score ≥9. The SOFA score is a marker for prognosis in patients with sepsis and shock and reflects the severity of multiorgan dysfunction. Medical files of the patients were reviewed for data collection by two trained investigators. Data were obtained on demographic, signs and symptoms upon hospital admission, comorbidities, smoking history, laboratory data, clinical management, lung computed tomography (CT) scan findings (pneumonia diagnosis was based on % of lung affected on CT scan imaging, according the grading: <10%, 10–50%; >50%), time from hospital admission to intubation, length of intubation, SOFA score, and outcome. The respiratory rate of the patients was recorded upon admission and immediately before intubation. 

All these demographic, clinical, and laboratory data were also evaluated according to the first and second COVID-19 waves in Brazil. The waves were defined according to a previous study from the Brazilian nationwide surveillance database for severe acute respiratory infections—Sistema de Informação de Vigilância Epidemiológica da Gripe (SIVEP-Gripe)—that described the first wave as being from April 2020 to epidemiological week (EW) 43 of 2020 (5 April 2020 to 24 October 2020), and the second wave from EW 44 of 2020 to the second EW of 2021 (8 November 2020 to 16 January 2021) [9].

### 2.2. Sample Collection and SARS-CoV-2 Detection

Samples of nasopharyngeal swab (NPS) were collected from all patients. NPS collection was performed by rubbing a swab on the posterior part of the nostril, with a rotating movement until the nasopharynx was reached, and the sample was obtained by rotating the swab gently for 5 s. Then, the swabs were placed into a 5 mL tube containing 3 mL of viral transport medium (VTM). The tubes containing the swabs were vortexed vigorously, and the samples were stored at −80 °C until RNA extraction. 

Viral RNA was extracted from 300 μL of NPS samples using the automatic MDX Biorobot ^®^ (Qiagen, Hilden, Germany). The detection of SARS-CoV-2 was performed using the molecular kit SARS-CoV-2 (Bio-Manguinhos/Fiocruz, Rio de Janeiro, Brazil) to detect E, N2 and human RNAse P targets, based on the Charité/Berlim protocol [31], according to manufacturer’s instructions. One-step real-time PCRs were carried out on the Taqman ABI 7500 Real Time PCR System^®^ (ThermoFisher, Waltham, MA, USA) using the following cycling conditions: 15 min at 45.0 °C, 2 min at 95 °C, followed by 45 cycles of 15 s at 95 °C and 30 s at 58 °C. Fluorescence was collected after each cycle at the annealing/extension step. All samples were run in duplicate. 

### 2.3. Statistical Analysis

All the statistical analyses were performed with the Statistical Package for Social Sciences version 20.0 (SPSS Inc., Chicago, IL, USA). Nonparametric tests were used for continuous variables since they did not present normal distribution (Shapiro–Wilk, *p* < 0.05). Frequencies and proportions were used for categorical variables. Spearman’s correlation was used to correlate COVID-19 viral load (through Ct value—cycle threshold) with the severity of disease (via SOFA and D-dimer). To associate the categorial variables of the study with the wave of COVID-19, we used Pearson’s chi-square or Fisher’s Exact tests. The Mann–Whitney test was applied to compare “COVID-19 Ct values” between waves. Binary logistic regression was used because the variable “wave” is a dichotomous categorical variable (first and second waves), and the variables cough, AST, hemoglobin, acute hepatitis, and use of corticosteroids were included in the model because they were statistically significant when evaluated separately in the comparison of the first and second waves, and they were included to predict the model. The significance level adopted was 5%. 

## 3. Results

### 3.1. Patient Cohort, Demographics, and Baseline Clinical Features

Between July 2020 and October 2021, 106 patients hospitalized in HUCFF with laboratory-confirmed SARS-CoV-2 infection were enrolled in this study: 67 (63.2%) were classified as non-severe COVID-19 cases and 39 (37.0%) as severe cases of COVID-19. All patients were followed during the period of hospitalization until the outcome (discharge or death). Demographics and baseline clinical features are outlined in Table 1. Most patients were female (50.9%), with a mean age of 62.82 ± 16.28 years (ranging from 17 to 95 years). The age of the patients was significantly higher in severe patients (*p* = 0.001). Twenty-nine patients were smokers (>20 packs/year), and 10.3% were immunodeficient. All patients had at least one comorbidity. Most reported comorbidities consisted of hypertension (78.3%), diabetes mellitus (34.9%), and cardiovascular diseases (27.3%). The frequency of underlying comorbidities was compared between groups, and a significantly higher proportion of neurological disease was observed in severe than in non-severe patients (OR 0.22, 95% CI [0.07–0.65]; *p* = 0.006) (Supplementary Table S1). No significant differences were observed regarding the presence of the remaining specific comorbidities between groups (Table 1).

### 3.2. COVID-19 Signs and Symptoms

Table 2 lists patient signs and symptoms upon hospital admission for both groups of patients with COVID-19. Common clinical symptoms of COVID-19 upon hospital admission included fever (45.2%), cough (54.7%), and dyspnea (56.6%). Dyspnea was observed in 71.8% of severe patients versus 47.7% of non-severe patients (OR 0.35; 95% CI [0.15–0.83; *p* = 0.024) (Supplementary Table S1). A small proportion of patients (28.3%) reported signs related to the central nervous system: dizziness (3.8%), headache (15.0%), and impaired consciousness (12.2%). Headache was diagnosed according to the International Classification of Headache Disorders (ICHD-3), and the patients included here fulfilled the criteria for the categorization of secondary headache, due to the fact that it occurred during the acute phase of the infection caused by SARS-CoV-2 [32]. However, no significant differences were observed regarding the presence of symptoms of the central nervous system between two groups. Gastrointestinal symptoms were rare (diarrhea (12.2%), vomiting (12.2%), and loss of appetite (10.3%)). 

### 3.3. Laboratory Data, Management, and Outcomes

Viral load was estimated by cycle threshold value (Ct). The mean Ct was significantly lower in severe patients (Ct = 24.6) than in non-severe patients (Ct = 28.6) (*p* = 0.01), indicating a higher viral load of SARS-CoV-2 among severe patients (Table 3). The Pearson correlations of SARS-CoV-2 viral load (Ct value) with D-dimer and SOFA score was tested for severe and non-severe patients, and the results showed a weak correlation for both variables, being positive for D-dimer (correlation coefficient = 0.093, *p* = 0.539) and negative for SOFA (correlation coefficient = −0.152, *p* = 0.172). However, patients with SOFA ≥ 9 (severe patients) were associated with a higher number of deaths. Of the 39 patients with severe COVID-19, 38 died (total mortality rate of 35.8%). Among the deaths, 37 (94.8%) occurred in patients with SOFA ≥ 9 (*p* < 0.001).

Various laboratory parameters, such as direct bilirubin (DB), protein C reactive, gamma–glutamyl transferase (GGT), lactate dehydrogenase (LDH), creatinine, ferritin peak, and neutrophil count, were significantly higher in the severe form of COVID-19 (*p* < 0.05) (Table 3). In addition, these laboratory parameters, together with D-dimer peak, were associated with COVID-19 mortality (*p* < 0.05) (Table 4).

COVID-19 treatment was largely symptomatic, in which corticosteroid (67.9%) was the most commonly used medication, mainly in severe rather than non-severe patients (100%, and 87.1%, respectively) (*p* < 0.05). Hemodynamic instability (the need to receive a vasopressor) was recorded in 39 (100%) severe patients, while most of the non-severe patients (89.5%) were stably hemodynamic during hospitalization (*p* < 0.05).

The average hospital admission to discharge (or death) time was 23.7 ± 2.93 and 18.5 ± 12.1 days, for severe and non-severe, respectively. Respiratory rate was significantly higher in severe patients than that in non-severe patents, while the mean peripheral oxygen saturation (SpO2) in severe patients was 0.89 (SD ± 0.08), which was significantly lower than that in the non-severe group (0.93%) (*p* < 0.05). Mechanical ventilation (or intubation) was required by all severe patients and by 13.43% (*p* < 0.05) of non-severe patients. Around 52% of the mildly ill patients required oxygen inhalation through a mask, high nasal oxygen flow inhalation or non-invasive ventilation (Table 5). 

Most of the patients showed pneumonia (51.8%; 55/106) on chest X-rays and chest computed tomography (CT). The clinical course of SARS-CoV-2-induced pneumonia displayed a broad spectrum of severity and progression patterns, since 42% of the patients developed severe pneumonia, with 10–50% of lungs affected. 

During hospitalization, 43.4% of the patients evolved acute renal failure, 37.7% acute myocardial infarction (AMI), 22.6% hepatitis, and 10.5% thrombotic events. The incidence rates of hepatitis, renal injury, and cardiac arrest were significantly higher in patients with severe COVID-19 compared with patients with mild symptoms (*p* < 0.05). Almost all severe patients died during hospitalization for COVID-19 (94.8%) (Table 5).

### 3.4. Demographic and Clinical Features of COVID-19 First and Second Waves Compared

We compared demographics, clinical features, severity (SOFA score), and mortality of COVID-19 hospitalized patients between the first and second waves. In our cohort, during the first wave of COVID-19, 35 (33%) patients were hospitalized, while 71 (67%) patients were hospitalized during the second wave of COVID-19. The incidence of severe pneumonia (10–50% or >50% of lung affected on chest X-rays and CT) increased from 37.1% in the first wave to 57.5% in the second wave, as well as the proportion of the patients who received respiratory support (22.9% vs. 39.4% non-invasive ventilation; 42.9% vs. 46.5% invasive mechanical ventilation) (*p* = 0.037). However, the proportion of deaths and severely ill patients (SOFA score > 9) did not differ significantly between the two waves. During the second wave, there were significantly fewer patients with the comorbidities of acute kidney injury and immunodeficiency than in the first wave (*p* < 0.05) (Table 6).

The most common symptom during the first wave was dyspnea (54.3%), while cough was statistically more prevalent during the second wave (*p* = 0.033) (Table 7). In general, most laboratory parameters evaluated were more altered during the second wave, mainly AST and hemoglobin (*p* < 0.05) (Table 8). During the second wave of SARS-CoV-2 infection, the incidence of acute hepatitis was higher than in the first one (*p* = 0.015) (Table 9).

There was no association between SARS-CoV-2 viral load (Ct value) and the outcome (death or discharge) (*p* = 0.197) (Table 4) or wave (*p* = 0.2) (Table 6). Regarding medication treatments, vasoactive amines were most commonly used in the first wave (42.9%), while corticoids were the significantly most used medications in the second wave (83.1% (*p* < 0.001)) (Table 9). A binary logistic regression was performed with the variables of cough, AST, hemoglobin, acute hepatitis, and use of corticosteroids. We observed that the use of corticosteroids was significant for the second wave (OR = 6.75; 95% CI = 2.42–18.82, *p* < 0.001) (Supplementary Table S1).

## 4. Discussion

COVID-19 impacted the health, societal, and economic landscape in unprecedented ways for more than 2 years [33]. A series of multivariable-adjusted analyses based on COVID-19 patient cohorts has reported that all ages of the population are susceptible to SARS-CoV-2 infection [34,35]. However, higher disease severity was found to be associated with certain demographic factors (such as older age and male gender) and with co-morbidities [36,37,38]. In our study, the median age of severe patients was higher than non-severe ones (59 years vs. 69 years). According to data from 79,394 confirmed cases in China [39], compared to patients aged 30−59 years, those aged below 30 and above 59 years were 0.6 (0.3−1.1) and 5.1 (4.2−6.1) times more likely to die after developing symptoms, respectively [39].

Among the comorbidities, neurological disease showed statistical significance with the severity of COVID-19 (*p* = 0.006). Of 18 studied patients with COVID-19 who had a history of neurological diseases/manifestations, 12 died. Among the neurological alterations, the most reported were previous stroke, followed by Alzheimer’s, polyneuropathy, and senile dementia. Studies have shown that about a third of COVID-19 patients develop neurological symptoms [40,41], in most cases associated with a more severe infection [40], indicating a potential neurotropism of SARS-CoV-2 as one of the possible mechanisms of neurological damage [42]. However, there are few data on the association of pre-existing neurological comorbidities and COVID-19 outcomes. A recent study evaluated the prevalence of pre-existing neurological comorbidities in a large cohort of patients diagnosed with COVID-19, finding that more than 20% of patients had neurological comorbidities, with cerebrovascular disease and cognitive impairment. Patients with pre-existing neurological diseases have been shown to have a significantly higher risk of infection (OR = 2.3), especially when associated with other comorbidities [43]. Tahira et al. (2021) showed that all-cause dementia, in particular AD, is a risk factor for severity and death in COVID-19 patients independent of age [44]. Furthermore, early involvement of the nervous system is related to more severe outcomes and a higher lethality rate, as shown in studies by our group and by other authors [45,46].

Upon infection, the most common symptoms were cough, fever, and fatigue, as has been frequently reported among COVID-19 patients [35,47,48]. However, dyspnea was more frequently reported in severe than in non-severe COVID-19 patients (71.8% vs. 47.8%, *p* < 0.001). Corroborating this finding, in a study with a cohort of 10,131 patients with COVID-19, those with dyspnea had a higher risk of hospitalization (aHR: 2.18; 95% CI: 2.02–2.36), mechanical ventilation (aHR: 2, 95; 95% CI: 2.49–3.49), and mortality (aHR: 1.78; 95% CI: 1.53–2.07) [49].

Patients with severe disease showed high viral load (Ct value < 25−27), while those with mild disease showed a medium viral load. Studies have suggested that SARS-CoV-2 is considered to have a high viral load with a Ct value < 25−27, medium viral load with a Ct value 25–32, and low viral load with a Ct value > 30–32 [50,51,52]. As reported by Liu et al., in this present study, the Ct values of severe patients with COVID-19 were significantly lower compared to those of non-severe patients at the time of admission, indicating that the mean viral load of severe patients was higher than that of mild patients [53]. There is controversy regarding the association between viral load and disease severity in patients with COVID-19 [54]. Some studies have claimed that viral load appears to be a poor predictor of disease outcome, and it is not age-dependent [55], while others found that exposure to high numbers of infective SARS-CoV-2 and kinetics of viral load were highly predictive markers of severe course and outcome in older patients [56,57]. In addition, a positive correlation between age (compared to >60 age) and peak viral load in patients with COVID-19 has been shown [50]. 

Abnormalities were found in laboratory biomarkers related to white blood cells (neutrophil counts), coagulation (peak of D-dimer levels), cellular injury (lactate dehydrogenase -LDH), inflammation (C-reactive protein), and renal (creatinine levels) and liver injuries (GGT, direct bilirubin and ALP levels). A significantly higher concentration of these biomarkers was associated with severity in the patients with COVID-19 enrolled in this present study. Similarly, previous findings have commonly associated these biomarkers with worse outcomes [11,58,59,60] and highlight their role in monitoring COVID-19 infection. Moreover, increases in LDH, ALP, CRP, and D-dimers had strong associations with mortality, in accordance with previous studies [61].

Increasing lactate dehydrogenase (LDH) has been linked to greater disease severity [34,37,62,63]. In the present study, we found higher levels of LDH in severe COVID-19 patients compared to non-severe COVID-19 patients. Recent data suggest that LDH may be related to respiratory function and be an important predictor of respiratory failure in COVID-19 patients. Moreover, corroborating this claim, in our study, patients with severe disease showed significantly lower oxygen saturation (SpO2) (<90) and higher respiratory rate (mean of 24.6) compared with mild patients, indicating an association between hypoxemia and worse clinical outcomes, which is similar to the findings of other studies [64,65]. A non-peer-reviewed study of 375 COVID-19 patients identified LDH and CRP thresholds (LDH < 365 U/L; high-sensitivity (hs) CRP < 41.2 mg/L) that reliably predicted a favorable prognosis. In our findings, critically ill patients had values above these thresholds (LDH mean of 514.8 U/L; CRP mean of 141 mg/L). Elevated levels of C-reactive protein (CRP) in COVID-19 patients indicate the occurrence of inflammation [66] and, alone or in conjunction with other biomarkers, have been proposed as a predictor of COVID-19 severity in other studies as well [62,67,68,69], and a positive correlation between elevated CRP levels and severely abnormal CT findings has been described [70].

In the present study, higher levels of ALP, IB, and GGT in severe patients when compared with non-severe patients indicate that liver injury can be a predictor factor for severity. A recent study described a syndrome of cholangiopathy in patients recovering from severe COVID-19 characterized by marked elevation in serum alkaline phosphatase (ALP) accompanied by evidence of bile duct injury upon imaging [71]. However, the involvement of COVID-19 in liver damage still remains unclear. 

Elevated D-dimer levels suggest extensive thrombin generation and fibrinolysis and are associated with poor prognosis in COVID-19 [72,73,74]; therefore, some studies have proposed the use of D-dimer blood levels for patient triage [75]. A study of 343 COVID-19 patients revealed that patients with D-dimer levels ≥2.0 μg/mL on admission had a hazard ratio of 51.5 (95% CI, 12.9–206.7) for death compared with patients who had levels <0.001 [76].

We investigated the sociodemographic and clinical characteristics of patients between the two waves of COVID-19. The main characteristics evaluated were symptoms, risk factors, and mortality. The mean age of patients infected during the first wave was lower than that of those infected during the second, although this difference was not significant. During the second wave of COVID-19, the incidence of severe pneumonia and the proportion of patients requiring invasive respiratory support (mechanical ventilation) increased, but the proportion of deaths did not differ significantly between the two waves. This finding may be due to an increase in the number of patients with hypoxemia during the second wave, suggesting a possible increase in severe cases during the second wave [9]. Another possibility could be the limited access of the patients to intensive care with availability of invasive respiratory support during the first wave. However, since the proportion of deaths did not differ significantly between the two waves, this may indicate more efficient care for COVID-19 patients admitted to the ICU during the second wave. Bastos [9] reported that in Brazil, the number of patients admitted to hospitals per week requiring respiratory support was 13,985 in the first wave, which increased by 192% during the second wave. Indeed, in a large study using data from the Influenza Epidemiological Surveillance Information System (SIVEP-Gripe), it was demonstrated that the second wave of the COVID-19 pandemic was more aggressive in Brazil compared with in other countries [10].

The most prevalent comorbidity in our data in both waves was hypertension, followed by diabetes, which is similar to the findings of other studies [10,77]. In the second wave, more individuals with COVID-19 developed acute hepatitis. Hepatic dysfunction has been seen in 14–53% of patients with COVID-19, particularly in those with severe disease [78]. Cases of acute liver injury have been reported and are associated with higher mortality [78,79]. Hepatic involvement in COVID-19 has been attributable to the direct cytopathic effect of the virus, an uncontrolled immune reaction or drug-induced liver injury, including lopinavir–ritonavir [80], tocilizumab [81,82], and remdesivir [78,83], and comorbidities. 

In our study, corticosteroid therapy was more used in the second wave (*p* < 0.05). A systematic review on the use of inhaled corticosteroids as an additional treatment option in COVID-19 found that corticosteroids probably reduce the risk of people admitted to hospital or death (admission to hospital or death before hospital admission), which may lower the number of days people have symptoms of mild COVID-19 and probably increase resolution of COVID-19 symptoms at day 14 [84]. However, they did not have enough evidence to know whether corticosteroids cause serious adverse events. Therefore, our findings raised a question of whether the increase rate of acute hepatitis during the second wave might be associated with prolonged use of corticosteroids in COVID-19 treatment. However, the role of classic immunosuppressive drugs, such as corticosteroids, in liver injury during the COVID-19 still needs more investigation.

Together, these findings suggest that the progression of the second wave resulted in an increase in severe cases, as has been reported in other countries such as the UK and Africa [24,85]. The epidemiological profile during the second wave may be due to the emergence of SARS-CoV-2 variants detected in Brazil, such as P.1 and P.2, considered variants of concern (VOCs) due to their public health impact. The increase in the prevalence of P.1 and P.2 among the sequenced variants coincides with the beginning of the second wave in Brazil [86,87,88]. The emergence of the Omicron (B.1.1.529) variant of SARS-CoV-2, in November 2021, the fifth VOC of SARS-CoV-2 [89], has posed high global public health threats, is the most mutated, is highly transmissible, and is comparatively resistant to immunotherapeutics or vaccines [89,90,91,92,93,94]. In this context, understanding the clinical and laboratory features of COVID-19 waves may help to improve strategies to counter Omicron variants, and so to overcome the challenges of the ongoing fourth COVID-19 wave. However, this study has a limitation regarding the sample size being limited, since only hospitalized patients able to sign the consent form were enrolled in the study. Furthermore, only those whose total laboratory parameters were collected in medical records were included in this study. Together, these restrictions limited the sample size.

## 5. Conclusions

The clinical and laboratory features of COVID-19 infection presented in this study corroborate those of previous studies, which together could be helpful in predicting disease severity. The identification of factors that predict complications of COVID-19 is pivotal for guiding clinical care, improving patient outcomes, and allocating scarce resources.

In this study, the first two pandemic waves of SARS-CoV-2 infection were compared. We found an increase in severe cases during the second wave; however, this was not reflected in an increase in deaths, suggesting a more efficient response of the health care system for COVID-19 patients admitted to the ICU during the second wave. Our evidence can contribute to understanding of the behavior of the COVID-19 pandemic in Brazil, helping to define public policies, allocate resources, and improve strategies for vaccination of priority groups.

## Figures and Tables

**Table 1:** Demographic and baseline clinical features of the study patients according to disease severity.

Parameter	Total (n = 106)	Non-Severe (n = 67)	Severe (n = 39)	*p*-Value ^a^
Characteristics				
Age (mean ± SD)	NA	59.1 ± 17.1	69.1 ± 12.5	0.001 *^,c^
Gender: Female—n(%)	54 (50.1)	33 (49.2)	21 (53.8)	0.7
Male—n(%)	52 (49.0)	34 (50.7)	18 (46.1)	0.7
Smoker ^b^—n(%)	29 (27.3)	19 (28.3)	10 (25.6)	0.8
Comorbidities				
Hypertension—n(%)	83 (78.3)	49 (73.1)	34 (87.2)	0.1
Diabetes—n(%)	37 (34.9)	22 (32.8)	15 (38.5)	0.7
Cardiac or cerebrovascular disease—n(%)	29 (27.3)	18 (26.8)	11 (28.2)	1.0
Chronic kidney disease—n(%)	23 (21.7)	14 (20.8)	9 (23.1)	0.8
Active Malignancy—n(%)	10 (9.4)	5 (7.4)	5 (12.8)	0.5
Cancer—n(%)	5 (4.7)	2 (2.9)	3 (7.7)	0.3
Thrombophilia—n(%)	5 (4.7)	3 (4.4)	2 (5.1)	1.0
Immunodeficiency or immunosuppression—n(%)	11 (10.3)	8 (11.9)	3 (7.7)	0.7
Neurological disease—n(%)	18 (16.7)	6 (8.9)	12 (30.8)	0.006 *
Respiratory disease—n(%)	8 (7.5)	5 (7.4)	3 (7.7)	1.0
Autoimmune disease—n(%)	6 (5.6)	5 (7.4)	1 (2.6)	0.4
Obesity—n(%)	6 (5.6)	3 (4.4)	3 (7.7)	0.7
HIV/Aids—n(%)	2 (1.8)	2 (2.9)	0	0.5
Liver disease—n(%)	12 (11.3)	5 (7.4)	7 (17.9)	0.1

n: number; 95% CI: 95% Confidence interval; NA: non-applicable; SD: standard deviation. * Statistical significance. ^a^ Pearson’s chi-squared test. ^b^ Smoker was considered someone who smokes more than 20 packs per year. ^c^ Mann–Whitney test.

**Table 2:** Patient signs and symptoms upon hospital admission, according to COVID-19 severity.

Signs and Symptoms	Total (n = 106)	Non-Severe (n = 67)	Severe (n = 39)	*p*-Value ^a^
General				
Cough—n(%)	58 (54.7)	39 (58.2)	19 (48.7)	0.4
Fever—n(%)	48 (45.2)	30 (44.7)	18 (46.1)	1.0
Malaise/fatigue—n(%)	43 (40.5)	28 (41.8)	15 (38.5)	0.8
Rhinorrhea—n(%)	5 (4.7)	3 (4.5)	2 (5.1)	1.0
Dyspnea—n(%)	60 (56.6)	32 (47.8)	28 (71.8)	0.02 *
Arthralgia—n(%)	1 (0.9)	1 (1.5)	0	1.0
Inappetence—n(%)	11 (10.3)	8 (11.9)	3 (7.7)	0.7
Diarrhea—n(%)	13 (12.2)	10 (14.9)	3 (7.7)	0.4
Emesis—n(%)	13 (12.2)	9 (13.4)	4 (10.2)	0.8
Sore throat—n(%)	3 (2.8)	3 (4.5)	0	0.3
Myalgia—n(%)	15 (14.1)	10 (14.9)	5 (12.8)	1.0
Any neurological symptom—n(%)	35 (33.0)	23 (34.3)	12 (30.8)	0.8
Central nervous system (CNS)—n(%)	30 (28.3)	20 (29.8)	10 (25.6)	0.8
Dizziness—n(%)	4 (3.8)	3 (4.5)	1 (2.6)	1.0
Headache—n(%)	16 (15.1)	12 (17.9)	4 (10.2)	0.4
Impaired consciousness—n(%)	13 (12.2)	6 (8.9)	7 (17.9)	0.2
Acute cerebrovascular disease—n(%)	3 (2.8)	1 (1.5)	2 (5.1)	0.5
Ataxia—n(%)	0	0	0	NA
Seizure—n(%)	4 (3.8)	2 (3.0)	2 (5.1)	0.6
Peripheral nervous system (PNS)—n(%)	13 (12.2)	7 (10.4)	6 (15.4)	0.5
Hypo/Ageusia—n(%)	9 (8.5)	5 (7.5)	4 (10.2)	0.7
Hypo/Anosmia—n(%)	7 (6.6)	6 (8.9)	1 (2.6)	0.2
Vision impairment—n(%)	1 (0.9)	0	1 (2.6)	0.4
Nerve pain—n(%)	0	0	0	NA
Peripheral neuropathy—n(%)	2 (1.9)	1 (1.5)	1 (2.6)	1.0
Skeletal muscle injury—n(%)	1 (0.9)	0	1 (2.6)	0.4

n: number; 95% CI: 95%Confidence interval; NA: non-applicable. * Statistical significance. ^a^ Pearson’s chi-squared test.

**Table 3:** Laboratory parameters of COVID-19 patients according to disease severity.

Parameter	Non-Severe (n = 67)	Severe (n = 39)	*p*-Value ^a^
Cycle threshold (Ct)—(mean ± SD)	28.6 ± 5.9	24.6 ± 7.6	0.01 *
Total bilirubin—median (range)	0.4 (0.0–5.0)	0.5 (0.2–3.1)	0.07
Direct bilirubin—median (range)	0.1 (0.0–1.3)	0.2 (0.1–2.1)	0.02 *
Indirect bilirubin—median (range)	0.3 (0.0–4.1)	0.3 (0.1–1.0)	0.6
C-reactive protein (CRP)—(mean ± SD)	97.9 ± 93.9	158.4 ± 111.2	0.005 *
CRP Peak—median (range)	140.5 (4.3–393.2)	294.5 (136.0–7911.0)	0.08
Alkaline phosphatase (ALP)—(mean ± SD)	106.1 ± 223.6	104.1 ± 73.2	0.9
Alanine aminotransferase (ALT)—median (range)	25.5 (5.0–249.0)	24.0 (8.0–181.0)	0.9
Creatinine kinase (CK)—median (range)	73,000.0 (0.0–387.0)	910,00.0 (17.0–2404.0)	0.3
Gamma-glutamyl transferase (GGT)—median (range)	60,500.0 (6.0–376.0)	104.0 (18.0–746.0)	0.03 *
Lactate dehydrogenase (LDH)—(mean ± SD)	355.2 ± 189.3	517.7 ± 323.5	0.02 *
Aspartate aminotransferase (AST)—(mean ± SD)	43.5 ± 30.2	49.2 ± 32.8	0.4
Blood urea nitrogen (BUN)—(mean ± SD)	60.7 ± 51.3	73.4 ± 40.8	0.2
Creatinine—median (range)	1000.0 (0.5–13.1)	1300.0 (0.5; 7.1)	0.01 *
Ferritin—median (range)	537.0 (39.0–3250.0)	644.0 (41.0–7189.0)	0.6
Ferritin peak—median (range)	796.0 (60.0–22,063)	1450.0 (12.9–70,183.0)	0.0008 *
Hemoglobin (Hb)—(mean ± SD)	11.9 ± 2.6	10.99 ± 2.5	0.07
White blood cells count (WBC)—median (range)	7400.0 (2200.0–29,390.0)	8400.0 (468,200–8400.0)	0.08
Basophiles—median (range)	0.0 (0.0–170.0)	0.0 (0.0–9364.0)	0.5
Eosinophiles—median (range)	0.0 (0.0–580.0)	0.0 (0.0–9364.0)	0.8
BANDS—median (range)	0.0 (0.0–3250.0)	0.0 (0.0–117050.0)	0.3
Neutrophiles—median (range)	5590.0 (3.1–51,255.0)	7264.0 (3168.0–107,606.0)	0.02 *
Lymphocytes—(mean ± SD)	1013.0 ± 731.2	1190.2 ± 954.6	0.3
Monocytes—median (range)	416.0 (1.0–1281.0)	510.0 (78.0–4682.0)	0.2
Platelets—(mean ± SD)	195,011.0 ± 126,933.0	157,103.0 ± 111,728.0	0.1
D dimer—(mean ± SD)	4584.8 ± 7186.7	2691.8 ± 2526.1	0.1
D dimer peak—(mean ± SD)	7004.0 ± 10,312.0	9630.0 ± 10,854.0	0.4

n: number; SD: standard deviation; NA: non-applicable. ^a^ Mann–Whitney test. * Statistical significance.

**Table 4:** Laboratory parameters of COVID-19 patients according to disease outcome.

Parameter	Survivors (n = 68)	Non-Survivors (n = 38)	*p*-Value ^a^
Cycle threshold (Ct)—(mean ± SD)	29.0 ± 1.0 (n = 25)	26.3 ± 1.8 (n = 22)	0.2
Total bilirubin—median (range)	0.5 (0.0–5.0)	0.5 (0.2–3.1)	0.2
Direct bilirubin—median (range)	0.1 (0.0–0.9)	0.2 (0.1–1.6)	0.07
Indirect bilirubin—median (range)	0.3 (0.0–4.1)	0.3 (0.1–1.0)	0.6
C-reactive protein (CRP)—(mean ± SD)	55.8 (0.9–294.0)	141.0 (1.2–460.0)	0.0008 *
CRP Peak—median (range)	135.0 (4.3–393.2)	315.0 (136.0–7911.0)	0.0001 *
Alkaline phosphatase (ALP)—(mean ± SD)	70.0 (11.0–1874.0)	85.0 (39.0–440.0)	0.04 *
Alanine aminotransferase (ALT)—median (range)	25.0 (5.0–249.0)	24.0 (8.0–181.0)	0.9
Creatinine kinase (CK)—median (range)	73.0 (18.0–387.0)	91.0 (17.0–2404.0)	0.5
Gamma-glutamyl transferase (GGT)—median (range)	61.0 (6.0–376.0)	101.5 (18.0–746.0)	0.04 *
Lactate dehydrogenase (LDH)—(mean ± SD)	341.13 ± 155.9	514.8 ± 325.7	0.003 *
Aspartate aminotransferase (AST)—(mean ± SD)	42723.0 ± 30.3	50,289.0 ± 32.7	0.2
Creatinine—median (range)	1 (0.5–13.1)	1.2 (0.5–6.9)	0.03 *
Ferritin—median (range)	515 (39.0–3250.0)	691 (41.0–79,462.0)	0.3
Ferritin peak—median (range)	866.0 (60.0–3931.0)	1692.5 (12.9–70,183.0)	0.002 *
Hemoglobin (Hb)—(mean ± SD)	11.8 ± 2.7	11.1 ± 2.4	0.1
White blood cells count (WBC)—median (range)	7900.0 (2900.0–21,860.0)	8400.0 (13,200.0–468,200.0)	0.2
Basophiles—median (range)	0 (0.0–170.0)	0 (0.0–9364.0)	0.9
Eosinophiles—median (range)	0 (0.0–580.0)	0 (0.0–9364.0)	0.9
Neutrophiles—median (range)	5510.0 (3.1–51,255.0)	7264.0 (3168.0–107,606.0)	0.01 *
Lymphocytes—(mean ± SD)	770.0 (2.7–4428.0)	980.0 (231.0–4682.0)	0.7
Monocytes—median (range)	416.0 (1.0–1920.0)	488.0 (78.0–4682.0)	0.6
Platelets—(mean ± SD)	182,000.0 (129.0–482,000.0)	161,000.0 (129.0–465,000.0)	0.1
D dimer—(mean ± SD)	1603.0 (1.1–34,507.0)	1703.0 (14.1–8064.0)	1.0
D dimer peak—(mean ± SD)	2008.0 (1.6–50,000.0)	6429.0 (255.0–48,436.0)	0.01 *

n: number; SD: standard deviation; NA: non-applicable. ^a^ Mann–Whitney test. * Statistical significance.

**Table 5:** Course of infection and complications.

Parameter	Total (n = 106)	Non-Severe (n = 67)	Severe (n = 39)	*p*-Value ^a^
COVID-19 wave 1—n(%)	35 (33)	22 (33)	13 (33)	1.0
COVID-19 wave 2—n(%)	71 (67)	45 (67)	26 (66)	1.0
Hospitalization time—(mean ± SD)	21.8 ± 20.4	23.7 ± 23.9	18.6 ± 12.1	0.1 ^b^
Respiratory rate at admission—(mean ± SD)	22.9 ± 5.6	21.9 ± 4.8	24.6 ± 6.6	0.03 *^,b^
O2 at admission (%)—(mean ± SD)	92 ± 0.06	90 ± 0.05	80 ± 0.08	0.03 * ^b^
Respiratory support days—(mean ± SD)	10.2 ± 9.0	9.7 ± 10.8	10.8 ± 6.1	0.57 ^b^
Non-invasive ventilation 1—n(%)	35	35 (52.2)	0	0.0001 *
Invasive ventilation 2—n(%)	48	9 (13.4)	39 (100)	0.0001 *
Without respiratory support—n(%)	22	22 (32.8)	0	0.0001 *
Pneumonia (<10%)—n(%)	1 (0.9)	1 (1.5)	0	1.0
Pneumonia (10–50%)—n(%)	45 (42.0)	31 (46.2)	14 (35.9)	0.31
Pneumonia (>50%)—n(%)	9 (8.5)	5 (7.5)	4 (10.3)	0.72
Hepatitis—n(%)	24 (22.6)	10 (14.9)	14(35.9)	0.01 *
Acute renal injury—n(%)	46 (43.3)	13 (19.4)	33 (86.8)	0.0001 *
Thrombotic event—n(%)	11 (10.3)	6 (8.9)	5 (12.8)	0.53
Heart failure—n(%)	40 (37.7)	2 (2.9)	38 (97.4)	0.0001 *
SOFA (highest of 3)—median (range)	10.2 (9.0–11.3)	2.7 (2.2–3.3)	12.4 (11.6–13.2)	0.0001 *^,b^

n: number; 95% CI: 95%Confidence interval; NA: non-applicable; SD: standard deviation. * Statistical significance. ^a^ Fisher’s exact test. ^b^ Mann–Whitney test.

**Table 6:** Demographic clinical features of COVID-19 according to the first and second waves.

Characteristics	First Wave (n = 35)	Second Wave (n = 71)	*p*-Value	Total (n = 106)
Age—(mean ± SD)	58.5 ± 18.5	64.9 ± 14.7	0.08 ^c^	NA
Cycle threshold (Ct)—(mean ± SD)	29.21 ± 7.8	26.16 ± 6.5	0.051 ^c^	NA
Gender: Male—n(%)	16 (45.7)	36 (50.7)	0.629 ^a^	52 (49.1)
Female—n(%)	19 (54.3)	35 (49.3)		54 (50.9)
Smoker: Yes—n(%)	8 (22.9)	21 (29.6)	0.465 ^a^	29 (27.4)
No—n(%)	27 (77.1)	50 (70.4)		77 (72.6)
Pneumonia on chest radiography: None—n(%)	22 (62.9)	30 (42.3)	0.091 ^a^	52 (49.0)
10–50%—n(%)	12 (34.2)	33 (46.5)		45 (42.5)
>50%—n(%)	1 (2.9)	8 (11.3)		9 (8.5)
O2 support: None—n(%)	12 (34.3)	10 (14.1)	0.037 ^a,^*	22 (20.8)
Nasal catheter—n(%)	8 (22.9)	28 (39.4)		36 (34.0)
Orotracheal intubation—n(%)	15 (42.9)	33 (46.5)		48 (45.3)
Outcome: Survivor—n(%)	22 (62.9)	45 (63.4)	0.958 ^a^	67 (63.2)
Non-survivor—n(%)	13 (37.1)	26 (36.6)		39 (36.8)
SOFA: <9 (mild to moderate)—n(%)	22 (62.9)	45 (63.4)	0.958 ^a^	67 (63.2)
>9 (severe)—n(%)	13 (37.1)	26 (36.6)		39 (36.8)
Comorbidities	27 (77.1)	56 (78.9)	0.839 ^a^	83 (78.3)
Hypertension—n(%)	27 (77.1)	56 (78.9)	0.839 ^a^	83 (78.3)
Diabetes—n(%)	11 (31.4)	26 (36.6)	0.598 ^a^	37 (34.9)
Cardiovascular disease—n(%)	10 (28.6)	19 (26.8)	0.844 ^a^	29 (27.4)
Chronic kidney disease—n(%)	14 (40.0)	9 (12.7)	0.001 ^a,^*	23 (21.7)
Chronic pulmonary disease—n(%)	8 (22.9)	13 (18.3)	0.581 ^a^	21 (19.8)
Neurological disease—n(%)	6 (17.1)	12 (16.9)	0.975 ^a^	18 (17.0)
Liver disease—n(%)	5 (14.3)	7 (9.9)	0.525 ^b^	12 (11.3)
Immunodeficiency or immunosuppression—n(%)	7 (20.0)	4 (5.6)	0.038 ^b,^*	11 (10.4)
Cancer—n(%)	4 (11.4)	6 (8.5)	0.722 ^b^	10 (9.4)
Autoimmune disease—n(%)	3 (8.6)	5 (7.0)	0.999 ^b^	8 (7.5)
Obesity—n(%)	0	6 (8.5)	0.175 ^b^	6 (5.7)
Thrombophilia—n(%)	3 (8.6)	2 (2.8)	0.329 ^b^	5 (4.7)
HIV/Aids—n(%)	2 (5.7)	0	0.107 ^b^	2 (1.9)

n: number; 95% CI: 95%Confidence interval; NA: non-applicable; SD: standard deviation. * Statistical significance. ^a^ Pearson’s chi-squared test. ^b^ Fisher’s exact test. ^c^ Mann–Whitney test.

**Table 7:** Description of signs and symptoms in patients with COVID-19.

Signs and Symptoms	First Wave (n = 35)	Second Wave (n = 71)	*p*-Value	Total (n = 106)
General				
Age—(mean ± SD)	58.5 ± 18.5	64.9 ± 14.7	0.08 ^c^	NA
Dyspnea—n(%)	19 (54.3)	41 (57.7)	0.735 ^a^	60 (56.6)
Cough—n(%)	14 (40.0)	44 (62.0)	0.033 ^a,^*	58 (54.7)
Fever—n(%)	18 (51.4)	30 (42.3)	0.372 ^a^	48 (45.3)
Malaise—n(%)	12 (34.3)	31 (43.7)	0.355 ^a^	43 (40.6)
Myalgia—n(%)	5 (14.3)	10 (14.1)	0.999 ^b^	15 (14.2)
Diarrhea—n(%)	5 (14.3)	8 (11.3)	0.755 ^b^	13 (12.3)
Emesis—n(%)	4 (11.4)	9 (12.7)	0.999 ^b^	13 (12.3)
Inappetence—n(%)	3 (8.6)	8 (11.3)	0.669 ^a^	11 (10.4)
Rhinorrhea—n(%)	1 (2.9)	4 (5.6)	0.526 ^b^	5 (4.7)
Sore throat—n(%)	1 (2.9)	2 (2.8)	0.999 ^b^	3 (2.8)
Arthralgia—n(%)	1 (2.9)	0	0.152 ^a^	1 (0.9)
Central nervous system (CNS)—n(%)	9 (25.7)	21 (29.6)	0.678 ^a^	30 (28.3)
Headache—n(%)	3 (8.6)	13 (18.3)	0.188 ^a^	16 (15.1)
Impaired consciousness—n(%)	6 (17.1)	7 (9.9)	0.348 ^b^	13 (12.3)
Dizziness—n(%)	0	4 (5.6)	0.300 ^b^	4 (3.8)
Seizure—n(%)	2 (5.7)	2 (2.8)	0.597 ^b^	4 (3.8)
Acute cerebrovascular disease—n(%)	1 (2.9)	2 (2.8)	0.999 ^b^	3 (2.8)
Peripheral nervous system (PNS)—n(%)	4 (11.4)	9 (12.7)	0.999 ^b^	13 (12.3)
Hypo/Ageusia—n(%)	4 (11.4)	5 (7.0)	0.474 ^b^	9 (8.5)
Hypo/Anosmia—n(%)	3 (8.6)	4 (5.6)	0.682 ^b^	7 (6.6)
Peripheral neuropathy—n(%)	0	2 (2.8)	0.999 ^b^	2 (1.9)
Vision impairment—n(%)	0	1 (1.4)	0.999 ^b^	1 (0.9)
Skeletal muscle injury—n(%)	0	1 (1.4)	0.999 ^b^	1 (0.9)

n: number; 95% CI: 95%Confidence interval; NA: non-applicable; SD: standard deviation. * Statistical significance. ^a^ Pearson’s chi-squared test. ^b^ Fisher’s exact test. ^c^ Mann–Whitney test.

**Table 8:** Laboratory parameters of COVID-19 patients according to the pandemic wave.

Parameter	n	Mean ± SD (Median)	% of Exams out of the Normality		*p*-Value
			First Wave	Second Wave	
Total bilirubin	80	0.7 ± 0.74 (0.5)	6.5	10.2	0.700 ^b^
Direct bilirubin	80	0.27 ± 0.35 (0.1)	64.5	81.6	0.085 ^a^
Indirect bilirubin	80	0.43 ± 0.52 (0.3)	3.2	6.1	0.999 ^b^
C-reactive protein (CRP)	104	283.72 ± 766.28 (215.20)	100	100	NA
Alkaline phosphatase (ALP)	105	105.36 ± 182.29 (79)	8.6	8.6	0.999 ^b^
Alanine aminotransferase (ALT)	105	34.85 ± 35.41 (24)	20.0	31.4	0.217 ^a^
Aspartate aminotransferase (AST)	105	45.63 ± 31.02 (38)	40.0	61.4	0.038 ^a,^*
Creatinine kinase (CK)	61	167.72 ± 323.71 (82)	28.6	23.1	0.639 ^a^
Gamma-glutamyl transferase (GGT)	105	115.45 ± 121.75 (66)	48.6	42.9	0.579 ^a^
Lactate dehydrogenase (LDH)	75	415.88 ± 258.28 (337)	66.7	75.0	0.440 ^a^
Blood urea nitrogen (BUN)	106	65.35 ± 47.92 (48.50)	97.1	91.5	0.421 ^b^
Creatinine	106	2.04 ± 2.58 (1.0)	42.9	25.4	0.067 ^a^
Ferritin	101	5064.03 ± 13,160.41 (977)	82.4	95.5	0.059 ^b^
Hemoglobin (Hb)	106	11.58 ± 2.61 (11.95)	68.6	46.5	0.032 ^a,^*
Leukocytes	106	13,566.13 ± 44,844.40 (7900)	28.6	36.6	0.411 ^a^
Basophiles	106	96.85 ± 909.00 (0)	0	1.4	0.999 ^b^
Eosinophiles	106	138.62 ± 912.24 (0)	0	2.8	0.999 ^b^
Metamyeolocytes	105	493.30 ± 5025.88 (0)	NA	NA	NA
Myelocytes	104	1305.56 ± 13,314.12 (0)	NA	NA	NA
BANDS	105	1290.25 ± 11,415.48 (0)	22.9	16.9	0.461 ^a^
Neutrophiles	106	8701.98 ± 11,426.54 (6332.5)	31.4	40.8	0.347 ^a^
Lymphocytes	106	1148.53 ± 894.78 (952)	40.0	45.1	0.620 ^a^
Monocytes	106	564.76 ± 548.40 (462)	8.6	16.9	0.376 ^b^
Platelets	106	183,287.63 ± 121,222.48 (179,000)	40.0	40.8	0.934 ^a^
D dimer	60	7950.37 ± 10,470.33 (4076)	92.6	97.0	0.583 ^b^

n: number; NA: non-applicable; SD: standard deviation. * Statistical significance. ^a^ Pearson’s chi-squared test. ^b^ Fisher’s exact test.

**Table 9:** Comparison of the complications and therapies used for COVID-19 during the two pandemic waves.

Complication	First Wave % (n)	Second Wave %(n)	*p*-Value	Total % (n)
Acute kidney injury	34.3 (12/35)	48.6 (34/70)	0.164 ^a^	43.8 (46/105)
Acute myocardial infarction	40.0 (14/35)	37.1 (26/70)	0.776 ^a^	37.7 (40/105)
Acute hepatitis	8.6 (3/35)	29.6 (21/71)	0.015 ^a,^*	22.6 (24/106)
Acute thrombotic event	5.7 (2/35)	12.9 (9/70)	0.329 ^b^	10.5 (11/105)
Medicines	First Wave % (n)	Second Wave %(n)	*p*-Value	Total % (n)
Corticosteroid	37.1 (13/35)	83.1 (59/71)	<0.001 ^a,^*	67.9 (72/106)
Anticoagulant	0 (0/35)	5.6 (4/71)	0.300 ^b^	3.8 (4/106)
Interferon	2.9 (1/35)	0 (0/71)	0.330 ^b^	0.9 (1/106)
Antiviral	2.9 (1/35)	0 (0/71)	0.330 ^b^	0.9 (1/106)
Antibiotic	5.7 (2/35)	0 (0/71)	0.107 ^b^	1.9 (2/106)

n: number; NA: non-applicable; SD: standard deviation. * Statistical significance. ^a^ Pearson’s chi-squared test. ^b^ Fisher’s exact test.

## Data Availability

The data are not publicly available due to ethical restrictions.

## Supplementary Materials

The following supporting information can be downloaded at: https://www.mdpi.com/article/10.3390/jcm12072568/s1, Table S1: Binary logistic regression of the significative variables.
