# Supplementary material for: Clinical Profile and Risk Factors for Severe COVID-19 in Hospitalized Patients from Rio de Janeiro, Brazil: Comparison between the First and Second Pandemic Waves
Source: J Clin Med. 2023 Mar 29;12(7):2568. doi: 10.3390/jcm12072568 (PMC10094970; doi:10.3390/jcm12072568)
Supplement: Supplementary file 1 [file jcm-12-02568-s001.zip › jcm-2162764-supplementary.pdf]

Clinical profile and risk factors for severe COVID-19 in patients hospitalized from Rio de Janeiro, Brazil: first and second epidemic waves compared

Luciane Almeida Amado, Wagner Luis da Costa Nunes Pimentel Coelho, Arthur Daniel Rocha Alves, Vanessa Cristine de Souza Carneiro, Otacilio da Cruz Moreira, Vanessa Salete de Paula, Andreza Salvio Lemos, Larissa Araujo Duarte, Elisa Gouvea Gutman, Fabricia Lima Fontes-Dantas , João Paulo da Costa Gonçalves, Carlos Henrique Ferreira Ramos, Carlos Henrique Ferreira Ramos Filho, Marta Guimarães Cavalcanti, Marisa Pimentel Amaro, Rafael Lopes Kader, Roberto de Andrade Medronho , Dmitry José de Santana Sarmento and Soniza Vieira Alves-Leon

Supplementary Table S1: Binary logistic regression of the significative variables

Variable	B	P-value	Odds Ratio (95% CI)
Present Cough	0.3	0.5	1.4 (0.5-4.0)
Altered AST	0.2	0.6	1.3 (0.5-3.5)
Altered Hemoglobin	-1.01	0.06	0.4 (0.1-1.1)
Acute Hepatitis	1.0	0.2	2.7 (0.7-11.2)
Corticosteroid use	1.9	<0.001	6.7 (2.4-18.8)

95% CI: 95%Confidence interval;
Hosmer and Lemeshow Test, p=294. The model classified 74.3% of cases.
